# Tourniquet Duration and Early Clinical and Biomarker Outcomes in Total Knee Arthroplasty: A Comparative Cohort Study

**DOI:** 10.3390/jcm15072675

**Published:** 2026-04-01

**Authors:** Nele Isabelle Pfeiffer, Jane Penelope Shaw, Alain Despont, Jelena Kummer, Rolf Spirig, Mai M. Abdelhafez, Emanuel Francis Liechti, Sandro Kohl, Frank Michael Klenke, Robert Rieben

**Affiliations:** 1 Department of Orthopaedic Surgery and Traumatology, Inselspital, Bern University Hospital, University of Bern, Freiburgstrasse 18, 3010 Bern, Switzerland; nele.i.pfeiffer@googlemail.com (N.I.P.); jelena.kummer@bluewin.ch (J.K.); emanuel.liechti@insel.ch (E.F.L.); sandro.kohl@hin.ch (S.K.); frank.klenke@insel.ch (F.M.K.); 2 Department for BioMedical Research, University of Bern, Murtenstrasse 24-28, 3010 Bern, Switzerland; janeshawboden@gmail.com (J.P.S.); alain.despont@unibe.ch (A.D.); maimoustafa@msa.edu.eg (M.M.A.); 3 CSL Behring AG, Wankdorfstrasse 10, 3014 Bern, Switzerland; rolfspirig@swissonline.ch; 4 Department of Pharmacology and Toxicology, Faculty of Pharmacy, October University for Modern Sciences and Art, Giza 12451, Egypt

**Keywords:** total knee arthroplasty, tourniquet, ischemia/reperfusion injury, pain, markers of inflammation

## Abstract

**Background**: Currently, the duration of tourniquet time in total knee arthroplasty is chosen by the surgeons and varies between 0 and 120 min. Studies evaluating the effect of tourniquet time in this surgery are heterogeneous, and there is limited information on molecular/complement profiling. The purpose of this study was, therefore, to determine whether the duration of tourniquet-induced limb ischemia during total knee arthroplasty influences reperfusion injury, resulting in pain, swelling, and the release of pro-inflammatory markers. **Methods**: In 40 patients undergoing total knee arthroplasty, a tourniquet was applied for up to 30 min (group A, short tourniquet) or 90–120 min (group B, long tourniquet). Postoperative pain and swelling served as primary outcome parameters. The levels of pro- and anti-inflammatory markers before surgery and 4 h, 24 h, and 48 h after surgery were used as secondary outcome parameters for exploratory testing. **Results**: There were no differences in numeric rating pain scale (NRS) scores and calf circumference between groups A and B. Patients in group B required patient-controlled intravenous analgesia more frequently than group A patients (47% versus 5%, group B vs. group A, *p* < 0.0001). In group B, a significantly higher increase in C3a and MIG levels between 4 h and 48 h, and a significantly higher increase for MIG and M-CSF between 24 h and 48 h, were observed. **Conclusions**: Tourniquet times between 90 and 120 min were not associated with higher pain levels or more swelling, but an increased need for intravenous analgesia and a higher increase in pro-inflammatory markers. This might be a consequence of a more pronounced ischemia/reperfusion injury with tourniquet times longer than 90 min.

## 1. Background

Total knee arthroplasty (TKA) is a surgery to replace the knee joint with a prosthesis, in the majority of cases due to osteoarthritis. This type of surgery can be done with the use of a tourniquet to restrict blood flow to the affected limb and, therefore, have better vision in the operative field, lower patient blood loss, decreased surgery time, and improved cementation [1,2,3]. However, several studies suggest a more pronounced swelling with higher pain levels, longer hospitalization, and a higher risk for thromboembolic events due to endothelial damage [4,5,6,7,8] with prolonged tourniquet application. The tourniquet can be applied for a short (only during cementation of the components, but not during the approach and preparation on the bones) or a long (during the whole procedure from opening the skin to wound closure) time period, and both are accepted in clinical practice with no consensus on benefits and harms. Several meta-analyses have addressed the harms and benefits of a tourniquet during TKA, but the results remain partly controversial. It is widely accepted that intraoperative blood loss is reduced [2,3,8,9,10] with tourniquet application, but its effect on postoperative pain is still not entirely clear. Some studies suggest increased short-term pain scores [2,9,10], but this finding is not always significant [8] or sometimes even inconsistent [3]. Furthermore, there seems to be a higher postoperative swelling after the use of a tourniquet [8,9], even though for this outcome, there is also a meta-analysis by Xu et al. showing conflicting data [3]. A multicenter comparison by Gibbs et al. did not find any differences between tourniquet use and no tourniquet use on the consumption of analgesics or postoperative complications [11]. Srivastava et al. published the latest guidelines on Surgical Management of Osteoarthritis of the Knee on behalf of the American Academy of Orthopedic Surgeons, stating with strong evidence that there is no difference in outcomes such as pain, function, and blood transfusions between the use and the non-use of a tourniquet [12]. This study was therefore conducted to clarify some of these contradictory results. The hypothesis was that a longer tourniquet time leads to higher pain levels and more swelling, in particular due to an aggravated ischemia/reperfusion injury (IRI). The latter would also lead to a more pronounced release of pro-inflammatory markers. Our study, therefore, included an exploratory analysis of a set of pro-inflammatory mediators, including growth factors, cytokines, and complement activation markers. The analyzed biomarkers included three molecules that are supposed to have protective effects in IRI: macrophage colony-stimulating factor (M-CSF), monokine induced by gamma interferon (MIG), and fetuin-A. However, whether an enhanced expression of these markers, both the pro- and anti-inflammatory ones, leads to long-term clinical consequences after TKA is still unclear to date.

Ischemia created by a tourniquet leads to IRI, a process that results in an increased release of pro-inflammatory factors after re-opening the tourniquet. A review conducted by Apichartpiyakul et al. in 2022 showed that after acute ischemia of the lower limbs due to embolism or thrombosis, IRI can lead to serious local complications, such as edema and subsequent necrosis of the skeletal muscle due to compartment syndrome [13]. In very rare but severe cases, systemic consequences can also occur, including acute respiratory distress syndrome (ARDS) or even multiorgan failure [13,14]. Those are associated with longer ischemia [14,15].

## 2. Patients and Methods

To assess the effect of tourniquet duration on IRI, a comparative single-blinded cohort study was performed, in which short (<30 min, group A) and long (90–120 min, group B) application of a tourniquet were compared in TKA. Group A served as the control group, and patients in group B served as cases. The hypothesis was that a longer tourniquet time leads to higher IRI.

The primary outcomes were pain and swelling. As secondary outcomes, the levels of pro- and anti-inflammatory markers in the plasma of the patients were investigated. Those markers were complement factor 3a (C3a), complement factor 5a (C5a), interferon-gamma induced protein 10 (IP10), macrophage inflammatory protein 1 alpha (MIP-1α), the soluble terminal complement complex sC5b-9, creatine kinase MM (CK-MM), D-dimers, thrombin–antithrombin complexes (TAT), complexes of plasminogen activator inhibitor 1 (PAI-I) with tissue-type plasminogen activator (t-PA), macrophage colony stimulating factor (M-CSF), monokine induced by gamma interferon (MIG), and fetuin-A. Blood loss served as an additional outcome. The study was approved by the local ethics committee and registered on ISRCTN.

To test our hypothesis, consent to participate was obtained from 40 patients scheduled for primary TKA in osteoarthritis (independent of the planned design of the prosthesis) at our hospital, between 2 May 2018 and 15 November 2021. Revisions and conversions were not included. There is currently no data available allowing a formal sample size calculation, so the number of participants was determined by the authors who planned the study. The trial ended when 40 patients were recruited. All eligible patients were asked by the surgeons who saw them at the consultations if they wanted to participate, or by telephone by one of the authors when they were scheduled for surgery. Inclusion criteria were age between 18 and 90 years with a planned TKA. Exclusion criteria were acute traumatic injury/infection, pregnancy/breastfeeding, patients who had elevated infection parameters one day before surgery (leukocyte count Lc > 10.5 × 10^9^/L, CRP > 10 mg/dL), and patients who took steroids or other medications that affect the immune system. If CRP (and/or Lc) were only moderately elevated (Lc < 12 × 10^9^/L, CRP < 15 mg/dL), a clinical assessment for infection was performed, since it is well known that osteoarthritis can lead to elevated CRP-levels [16,17]. If this showed no evidence of infection, elevated Lc and CRP were interpreted as being caused by the osteoarthritis, and these patients were included in the study. Patients were then randomized electronically into two groups right before surgery: Group A with a short tourniquet time (max. 30 min, only during cementation) and group B with a long tourniquet time (90–120 min, during the whole surgery). For the tourniquet, the Automatic Tourniquet System ATS 4000, Zimmer, Warsaw, IN, USA, was applied. Right before surgery, the surgeon was handed a sealed envelope with the assigned group of the patient and used the tourniquet accordingly. This envelope was stored in the private office of one of the authors who designed the study, and it was handed out by him or the first author. The surgeon determined whether the tourniquet was inflated to 350 mmHg or to systolic blood pressure plus 100 mmHg; there was no predefined protocol regarding this choice. This procedure was the same for long and short tourniquet applications. The surgery team was not blinded to long or short tourniquet application since this is technically not feasible. Patients, hospital staff, and laboratory staff outside the surgery were blinded since they had no access to the assignment of the groups. All procedures, except for the duration of the tourniquet, did not differ between the groups. Patients could choose with the anesthesiologist if they preferred general or spinal anesthesia. All eligible patients were given 1 g of tranexamic acid intravenously (no prior thromboembolic events, and sufficient renal function) during anesthetization, and all patients received Enoxaparine 40 mg subcutaneously (Clexane) once daily, starting 6 h postoperatively, as venous thromboembolism (VTE) prophylaxis. Intraoperative fluid and temperature management were done by the anesthesiologists; there were no predefined protocols on temperature and fluid management.

Blood samples were collected and the calf circumference was measured before surgery and 4 h, 24 h, and 48 h after surgery, when routine blood samples were taken from the patients. Nurses who attended to the patients took the blood samples, measured the calf circumference, and asked patients about their pain score. After taking the blood samples, they called the person responsible for sample preparation from the laboratory. Samples were then directly collected and taken to the laboratory, where they were centrifuged and stored at −70 °C. Measuring the calf circumference was always done 15 cm underneath the distal patella pole. The edges of the measuring tape were marked with a permanent marker at the first measurement and reused for the following ones. Pain scores were always noted in the medical chart; the calf circumference was noted on predefined tables. No predefined standard analgesic protocol was used postoperatively, even though all patients were routinely prescribed Oxycodone/Naloxone 10 mg/5 mg for pain management after surgery. The prescription of other pain medications (Paracetamol, Metamizole, NSAIDs, and Tramadol) depended on patients’ age, renal function, and allergies. In reserve, there was Oxycodone 5 mg every 4 h for every patient. If patients reported unbearable pain after having made full use of their reserve pain medication, PCIA was installed by the anesthesiologist on call. Nurses, as well as the laboratory staff performing the biomarker analytics, were blinded. Data collection ended 48 h after surgery.

The following clinical data were collected of each patient: sex, age (at the day of surgery), body mass index (BMI), American Society of Anesthesiologist classification (ASA-score), pain scores before and after surgery (numeric rating scale NRS 0 to 10 after 4 h, 24 h, and 48 h), duration of surgery, systolic blood pressure before tourniquet inflation, tourniquet pressure, tourniquet time, intraoperative blood loss (volume in the aspirator minus saline), application of tranexamic acid, the need for patient-controlled intravenous analgesia (PCIA), and calculated equipotent doses of per os morphine and PCIA.

To evaluate the markers of IRI, the following laboratory data were collected for each patient: D-dimers, C5a, TAT, fetuin-A, PAI-1/tPA complexes, CK-MM, and C3a by ELISA; IP10, M-CSF, MIG, MIP-1α, and sC5b-9 were quantified with a Bio-Plex assay from Bio-Rad. For patients 1–20, the following additional markers were measured in plasma: PDGF, TNF-alpha, MCP-1, G-CSF, GM-CSF, CTACK, Eotaxin, GRO-alpha, HGF, IFN-alpha2, IFN-gamma, IL-1ra, IL-2Ralpha, IL-1beta, IL-6, IL-7, IL-8, IL-9, IL-13, IL-16, IL-18, LIF, MIF, MIP-1beta, RANTES, SCF, SCGF-beta, SDF-1alpha, TNF-beta, TRAIL, bFGF, and VEGF with the Bio-Plex assay from Bio-Rad. Randomization was done in two groups of 10 case patients and 10 control patients each. This allowed a first analysis of the exploratory data on the inflammatory markers and, therefore, to narrow the set of analyses for the second group. The concentration of the markers was calculated by creating a standard curve using GraphPad Prism (Version 10) from the ELISA. Concentrations of the markers measured with the Bio-Plex multiplex suspension array technique were calculated directly by the machine based on a 5-parameter logistic regression algorithm. All randomized participants were included in calculations.

We compared epidemiological data using Student’s *t*-test, one-way ANOVA for multiple comparisons, and a contingency table for categorical data. For blood loss, since data did not show a Gaussian distribution, the Mann–Whitney U-test was used. Because of the quite different baseline levels in our patients, who had underlying diseases and were often elderly, we calculated the increase in pro-inflammatory markers over time, expressed as the slope between data points. To obtain the x-fold increase or decrease compared to baseline (BL) before surgery, all data were normalized by division using the BL values. The same calculations were then done with normalization to the 4 h values to check for later increases or decreases. To take the dynamics of our data into account, the linear slope of the graphs from the 4 h to the 48 h sample (without 24 h) was assessed and normalized to BL, and the same was done for the 24 h to the 48 h sample, normalized to 4 h. To check for differences, the calculated slopes were compared using Student’s *t*-test, with *p* ≤ 0.05 considered as statistically significant. Missing data occurred during data collection and was completely at random; calculations were done without those values.

For pain medication, the doses of opioids from immediately postoperative to 48 h after surgery were totaled. Intraoperative medication during anesthesia was not considered. Equipotent doses of per os morphine and PCIA were calculated and added up to compare the different patients with each other. The dose was multiplied by 1.5 for Oxycodone and by 11 for intravenous hydromorphone from the PCIA [18]. Other pain medications were not administered during the observation period of this study due to allergies or intolerances and restrictions on non-steroidal anti-inflammatory drugs (NSAIDs) in older patients and in patients with reduced renal function.

All procedures were carried out in alignment with the ISCRTN-registration, except for the following: initially, it was planned to measure the circumference, register the pain score, and take blood samples directly after the operation. However, this was technically not feasible since patients need the most care right after surgery. Asking pain scores was also not feasible in patients with complete anesthesia. Furthermore, the target sample size at registration was 60 patients. Due to the COVID pandemic, recruitment took much longer than suspected because TKA is not a vital surgery, and these operations were canceled for prolonged time periods during the pandemic. The sample size was therefore lowered to 40 patients.

## 3. Results

There were 39 TKAs and one unilateral knee arthroplasty (UKA). The UKA was included since it was scheduled for TKA, but the decision for UKA was made intraoperatively due to better unicompartimental cartilage than suspected. The surgical approach and setup did not differ from the TKA. All interventions were done at the Department for Orthopedic Surgery, University Hospital/Inselspital Bern, Switzerland, by four different surgeons. All 40 patients were included in the analyses. Further information is shown in the CONSORT checklist and the flow diagram (Supplementary File S1a,b).

The tourniquet time differed significantly between group A (23 patients), the short tourniquet group, and group B (17 patients), the long tourniquet group. There were no differences between the groups regarding age, sex, baseline health status, duration of surgery, type of anesthesia, tourniquet pressure, and the application of tranexamic acid, as shown in Table 1 and Table 2. Further information with detailed values by patient of duration of surgery, tourniquet pressure, and tourniquet duration is available in Supplementary File S2. No harm was identified during the follow-up of 48 h.

**Figure 1:**
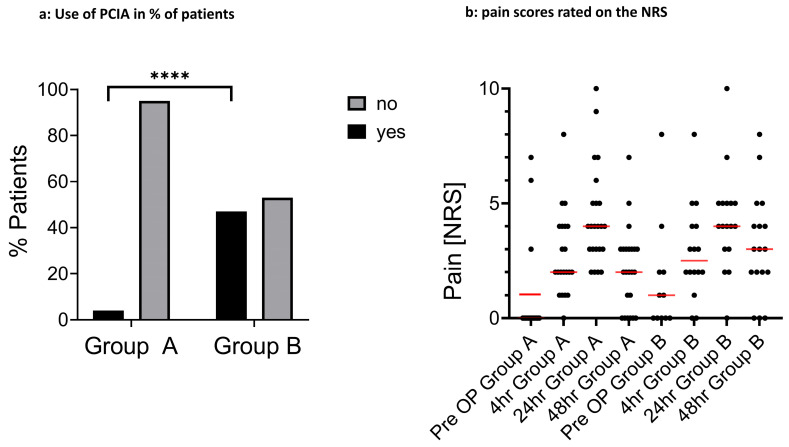
Use of PCIA in % of patients and pain scores rated on the NRS. (**a**) Use of PCIA in % of patients. The *p*-value was calculated by Fisher’s exact test. ****: significant *p*-value (*p* < 0.001) (**b**) Pain rated on the NRS between 0 (no pain) and 10 (worst pain imaginable), with red lines showing mean values. Each dot represents one patient. Different numbers of dots are due to missing pain score values. The *p*-values were calculated by one-way ANOVA.

Primary outcome parameters: Pain was evaluated as the first primary outcome parameter. There were no significant differences between NRS pain scores for group A and group B. Pain scores increased until 24 h after surgery, with a mean NRS of 4 in both groups, and they started to decrease afterwards to NRS 2 in group A and NRS 3 in group B. However, patients in the long tourniquet group (group B) needed patient-controlled intravenous analgesia (PCIA) more often than patients from group A (group A: 5%, group B: 47%, *p* < 0.001, Figure 1a,b). The total amount of morphine equivalent for each group can be found in Supplementary File S3.

**Figure 2:**
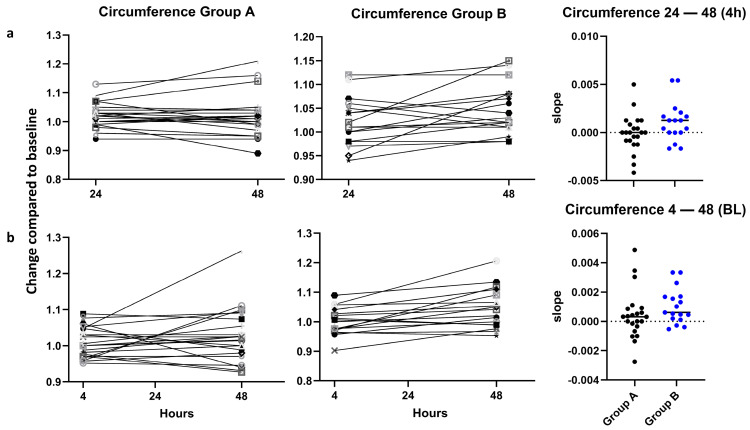
Increase in the calf circumference at different time points. (**a**) Increase in calf circumference between 24 and 48 h (normalized to 4 h), *p* = 0.07. (**b**) Increase in calf circumference between 4 h and 48 h normalized to BL, *p* = 0.21, both calculated by unpaired *t*-test. Each symbol represents an individual patient. For better identification, dots of group A are black and dots of group B in blue.

For swelling, a non-significant tendency (*p* = 0.07) was found for a higher increase in the calf circumference from 24 h to 48 h in group B compared to group A when the values were normalized to 4 h (Figure 2a). There was no significant difference in swelling over the whole period of 4–48 h, with the values normalized to the BL (Figure 2b).

**Figure 3:**
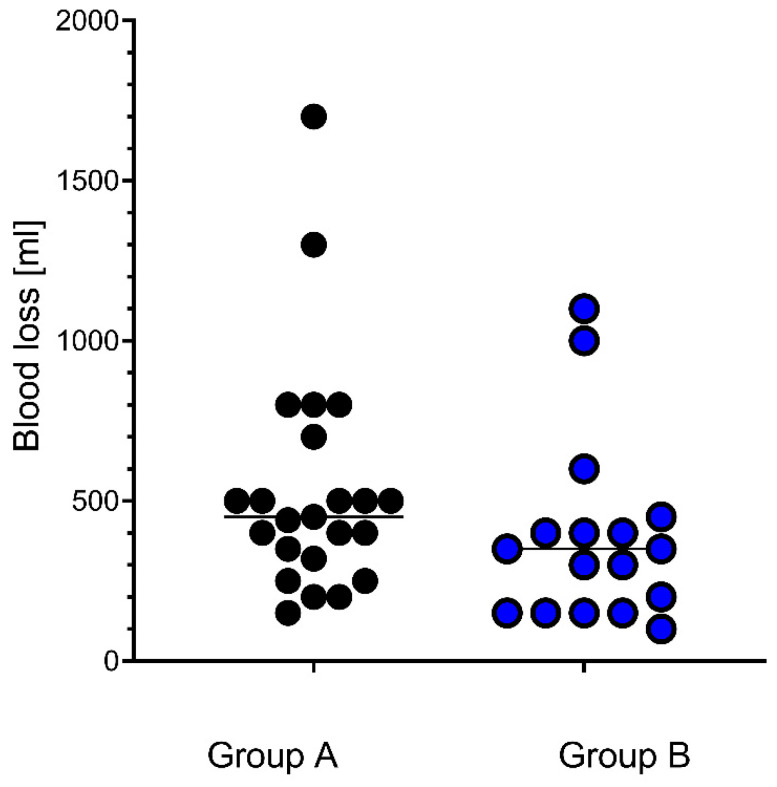
Intraoperative blood loss. Blood loss of group A was 540 mL, and blood loss of group B was 385 mL; each dot represents an individual patient, with lines showing the median values. *p* = 0.046, calculated by the Mann–Whitney U-Test. For better identification, dots of group A are black and those of group B in blue.

Blood loss was lower in group B with the long tourniquet than in group A, with a *p*-value of 0.046 (Figure 3).

Effect estimates with 95% CIs for primary outcomes (pain, swelling, and blood loss) can be found in Supplementary File S4.

**Figure 4:**
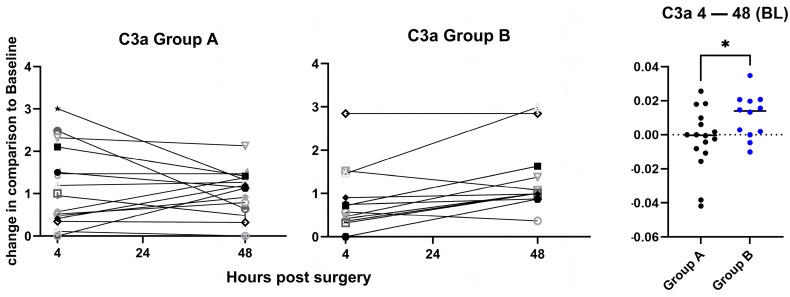
Change in C3a plasma levels. Changes in C3a plasma levels between 4 h and 48 h after the operation are shown for group A (**left panel**) and group B (**middle panel**). Values are fold changes normalized to baseline (BL). The slopes for the change between 4 h and 48 h are plotted on the (**right panel**), with indication of the median values, showing a significantly higher increase in the C3a levels in group B as compared to group A, and *p* = 0.039, calculated by unpaired *t*-test. Each symbol represents an individual patient. BL: Baseline. *: *p*-value significant.

Secondary outcomes: All biomarker analyses were exploratory and not adjusted for multiple comparisons. A significantly higher mean increase in C3a levels between 4 h and 48 h was found in group B when values were normalized to BL (Figure 4). In group A, C3a levels decreased with a mean slope of −0.003, whereas in group B, they were still increasing postoperatively with a mean slope of 0.011. There were no significant changes between 24 h and 48 h when values were normalized to 4 h (Supplementary File S5).

**Figure 5:**
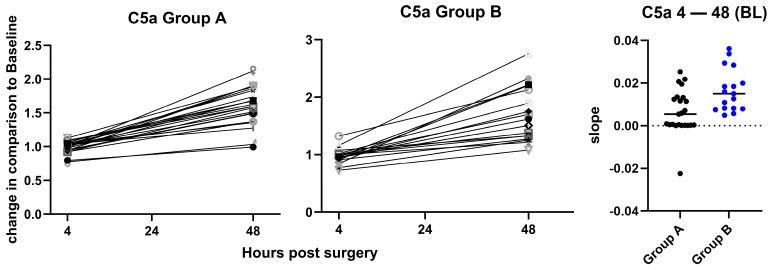
Change in C5a plasma levels. Changes in C5a plasma levels between 4 h and 48 h after the operation are shown for group A (**left panel**) and group B (**middle panel**). Values are fold changes normalized to baseline (BL). The slopes for the change between 4 h and 48 h are plotted on the (**right panel**) with indication of the median values. Plasma levels of C5a were consistently increased at 48, but the slopes were not significantly different between the groups, and *p* = 0.23, calculated by unpaired *t*-test. Each symbol represents an individual patient. BL: Baseline.

In contrast, for C5a, a postoperative increase in the plasma levels between 4 h and 48 h, normalized to BL, was observed in both groups, but no significant inter-group differences were found (Figure 5). This increase was less pronounced between 24 h and 48 h when normalized to 4 h, with no significant inter-group differences (Supplementary File S6).

**Figure 6:**
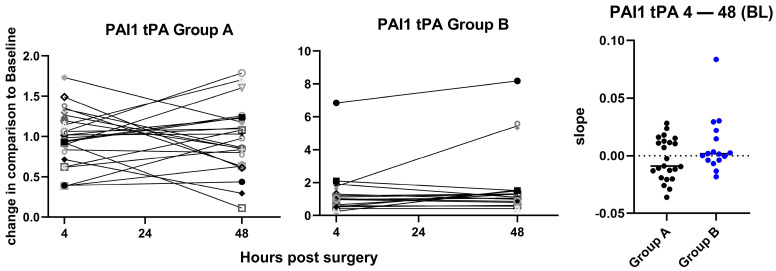
Changes in PAI-1/tPA complex plasma levels. Changes in the plasma levels of PAI-1/tPA complexes between 4 h and 48 h after the operation are shown for group A (**left panel**) and group B (**middle panel**). Values are fold changes normalized to baseline (BL). The slopes for the change between 4 h and 48 h are plotted on the (**right panel**) with indication of the median values, showing a non-significant tendency for a higher increase in the PAI-1/tPA levels in group B as compared to group A, and *p* = 0.09, calculated by unpaired *t*-test. Each symbol represents an individual patient. BL: Baseline.

PAI-1/tPA complexes were measured to assess endothelial cell activation due to IRI. As shown in Figure 6, the changes in PAI-1/tPA complex levels from 4 h to 48 h post-surgery were not significantly different between groups, but a non-significant tendency for a higher increase in PAI-1/tPA complexes, suggesting an increased procoagulant state of the endothelium, was found in group B compared to group A (Figure 6). There was no difference between 24 h and 48 h when normalized to 4 h (Supplementary File S7).

There were no differences in the levels of Fetuin A, TAT, CK-MM, and D-Dimers between groups A and B from 4 h to 48 h post-surgery and from 24 h to 48 h post-surgery. Graphs are available in Supplementary Files S8–11.

**Figure 7:**
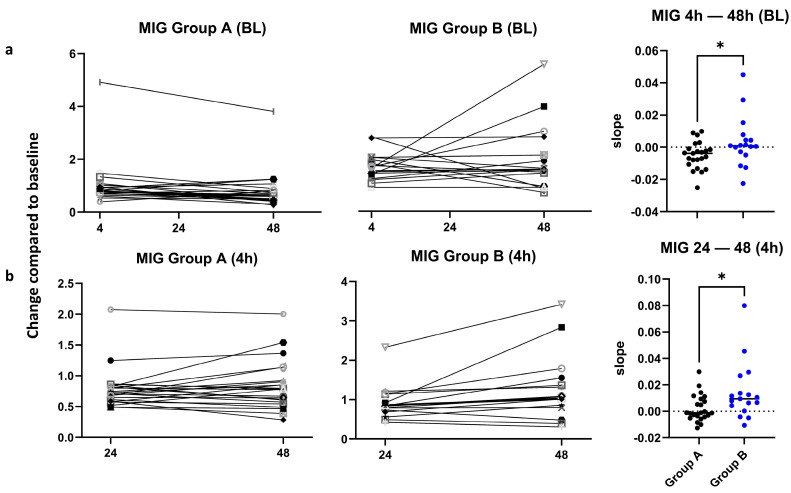
Change in MIG plasma levels. (**a**) Changes in the plasma levels of MIG between 4 h and 48 h after the operation are shown for group A (**left panel**) and group B (**middle panel**). Values are fold changes normalized to BL. The slopes for the change between 4 h and 48 h are plotted on the (**right panel**) with indication of the median values, showing a significantly higher increase in the MIG levels in group B as compared to group A, *p* = 0.036. (**b**) Changes in the plasma levels of MIG between 24 h and 48 h after the operation, normalized to 4 h, analogously to part a. *p* = 0.02, calculated by unpaired *t*-test. Each symbol represents an individual patient. BL: Baseline. *: *p*-value significant.

In the Bio-Plex assay for pro- and anti-inflammatory markers, a significantly higher increase for MIG was shown in group B, both between 4 h and 48 h (normalized to BL) and between 24 h and 48 h (normalized to 4 h, Figure 7). The values slightly decreased between 24 h and 48 h in group A, whereas an increase in the values was seen for group B. The difference in the average calculated slopes was significant, with *p* = 0.036 (Figure 7a). There was also a significant inter-group difference for MIG in the time frame 24 h to 48 h, normalized to 4 h, with *p* = 0.02 (Figure 7b).

**Figure 8:**
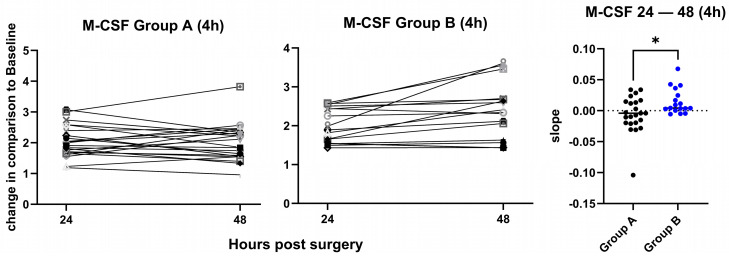
Change in M-CSF plasma levels. Changes in the plasma levels of M-CSF between 24 h and 48 h after the operation are shown for group A (**left panel**) and group B (**middle panel**). Values are fold changes normalized to 4 h. The slopes for the change between 24 h and 48 h are plotted on the (**right panel**) with indication of the mv, showing a significantly higher increase in the M-CSF levels in group B as compared to group A, and *p* = 0.019, calculated by unpaired *t*-test. Each symbol represents an individual patient. *: *p*-value significant.

M-CSF levels, normalized to 4 h, increased more significantly between 24 h and 48 h in group B than in group A, *p* = 0.019 (Figure 8). M-CSF levels when normalized to BL showed no significant increase or decrease (Supplementary File S12).

There were no significant differences between groups in the changes over time for IP-10, MIP-1α, and sC5b-9. Calculations and graphs are available in the Supplementary Files S13–S15. Additionally, CTACK, eotaxin, bFGF, G-CSF, GM-CSF, GRO-alpha, HGF, IFN-alpha2, IFN-gamma, IL-1beta, IL-1ra, IL-2Ralpha, IL-6, IL-7, IL-8, IL-9, IL-13, IL-16, IL-18, LIF, MIF, MIP-1beta, RANTES, SCF, SCGF-beta, SDF-1alpha, TNF-beta, TRAIL, MCP-1, PDGFbb, and VEGF did not show any significant differences for patients 1–20 between group A and group B in the one-way ANOVA when normalized to BL, so that we did not investigate those for patients 21–40. Graphs are available in Supplementary File S16. All raw concentrations of all the examined markers are available in Supplementary File S17.

## 4. Discussion

Pain levels reported by the patients themselves on an analog pain scale (NRS) were not significantly different between the two groups (Figure 1b). However, patients in the long tourniquet group (group B) more frequently used patient-controlled intravenous analgesia (PCIA) (Figure 1a). The reason for this might be higher pain levels in patients in group B with long tourniquet times, which were successfully lowered by PCIA. The need for PCIA, however, allows no direct inference to be made about significantly higher pain levels, especially since there was no predefined analgesics protocol.

For swelling, measured as the increase in the calf circumference, the differences between short and long tourniquet duration were not statistically significant either, but showed a tendency for increased swelling with long tourniquet application 24 h to 48 h post-operation. Our interpretation of this finding is that from day 1 to day 2, there might be further progress in the swelling after use of a long tourniquet time compared to use of a short tourniquet time. This would be in line with a meta-analysis by Han et al. and two randomized control trials, which found higher pain scores, more swelling, and also worse functional scores with longer tourniquet application [6,9,19]. One of those RCTs compared a non-tourniquet group to a tourniquet group in robot-assisted TKA, and the second one compared a non-tourniquet to a tourniquet group in ankle surgery, which limits comparability to this study. However, a big meta-analysis by Xu et al. could not find any significant differences in pain, swelling, and range of motion [3], so the influence of the tourniquet on those outcomes is not entirely clear. One limitation of this meta-analysis is the large heterogeneity in its included studies.

Intraoperative blood loss was significantly lower in the long tourniquet group due to reduced bleeding by compression of blood vessels, which is one of the reasons to use a tourniquet. However, it is unclear if a reduced blood loss in the long tourniquet group has any advantages for the patients, considering the need for transfusions or a drop in hemoglobin. Several studies and meta-analyses also showed lower intraoperative blood loss with longer tourniquet application [3,6,9,19]. Measuring blood loss in these studies was heterogeneous, ranging from measuring postoperative hemoglobin to estimating from the increase in gauze weight and the volume minus saline in the aspirator bottle. In this study, only the amount minus saline in the aspirator bottle was the estimated blood loss, but the tendency for a lower intraoperative blood loss in the long tourniquet group remains, regardless of the way of measuring it. None of these studies could show an impact on the need for blood transfusions.

For the analyzed inflammation markers, there are no prior studies comparing those markers in TKA between a short and a long tourniquet duration. In this study, there was a significantly higher expression of C3a in the long tourniquet group. C3a is a central marker for complement activation via any of the three initiation pathways. Complement activation is known to occur in the context of IRI, and our data support the hypothesis that the longer tourniquet time possibly leads to a more pronounced IRI [20,21,22]. However, those studies investigated C3a in free flaps, renal IRI, and liver transplants. When comparing the increase in the inflammation markers M-CSF and MIG between 24 h and 48 h, normalized to the values at 4 h, significantly higher values for both markers in the long tourniquet group were found. In addition, a (non-significant) tendency for a higher increase was shown for PAI-1/tPA complexes and a significant increase in MIG between 4 h and 48 h, normalized to the values at BL. Elevated levels of PAI-1/tPA complexes are indicative of a procoagulant state of the endothelium, which may cause clinical problems due to hypercoagulation. The latter has already been described by Watanabe et al. [23], who showed that patients with thrombus after TKA with a tourniquet had higher PAI-1/tPA and D-Dimer levels 30 and 90 s post-surgery. Those levels decreased quickly. This might be the reason why our study showed no significant differences in D-dimer levels, since the earliest sample was taken 4 h after TKA. An increased pro-thrombotic state of the endothelium might also result in a higher risk of deep vein thrombosis after TKA with a long tourniquet, as shown by Huang et al. [24].

There were also increased levels of M-CSF and MIG in the long tourniquet group, which are rather protective factors in IRI. M-CSF is increased during inflammation in a kidney ischemia model in mice [25,26] and leads to enhanced revascularization and, therefore, an equally enhanced regeneration of skeletal muscle after ischemia [27]. MIG binds competitively to certain glycosaminoglycan receptors and hereby inhibits the chemotaxis of several chemokines. This leads to reduced tissue injury and inflammation in mouse models [28,29]. The results of our study, therefore, suggest that increased IRI in the long tourniquet group possibly leads to an upregulation of the tissue-protective and regenerative factors M-CSF and MIG, particularly between 24 h and 48 h after surgery, as shown in previous mouse models.

The results of this study show that the surgeon should always critically evaluate the standard use of a tourniquet in TKA. Potential risks (higher need for analgesics, higher pain scores, and more pronounced swelling due to an aggravated IRI with its corresponding markers) and its advantages (lower intraoperative blood loss, better vision of the operative field, and better cementation), however, need to be further confirmed in future studies. Whether our findings, however, also have an impact on long-term outcomes (longer than 48 h) remains unclear, which necessitates the need for studies focusing on long-term outcomes. There also seems to be a need for standardized protocols regarding when the use of a tourniquet would be beneficial for a patient, which is another point future research could focus on.

## 5. Limitations

This study has some limitations. All quantifications of pro-inflammatory markers, growth factors, cytokines, and complement activation were done from the blood serum of patients and not the tissue of the affected limb. Furthermore, there was a relatively small number of patients in each group, limiting the power of this study. Considering blood loss, the need for transfusions or a significant drop in hemoglobin was not monitored. In this study, only the volume minus saline in the aspirator bottle was the estimated blood loss, leading to the possible underestimation of intraoperative blood loss. Furthermore, there was a certain heterogeneity in our study: the type of anesthesia (total vs. spinal), the use of tranexamic acid, variability in tourniquet pressure, and the UKA, which was included. Another limitation is the lack of a standardized postoperative analgesia protocol. Measurements of the calf circumference were done with a measuring tape on marks applied during the first measurement. This type of measurement is not very accurate.

Our follow-up was 48 h, which neglects possible later side effects or benefits. Furthermore, biomarkers were investigated exploratively, which limits the generalizability of the findings. Calculations of the slope represent a simplified longitudinal analysis. Future studies might consider using more advanced modeling techniques.

## 6. Conclusions

Our results show a higher need for PCIA in the long tourniquet group, suggesting higher pain levels when applying a tourniquet for 90–120 min, but no significant difference in monitored pain levels or swelling. However, one advantage of using a tourniquet is a reduction in intraoperative blood loss. Additionally, we found higher levels of pro-inflammatory markers, which we investigated exploratively. Their influence on clinical outcomes remains unclear. In our opinion, though, this shows that the application of a long tourniquet can lead to higher IRI. Our results cannot be transferred to long-term outcomes due to the relatively short follow-up of 48 h.

Even though TKA is done more and more often without the use of a tourniquet, the conclusions of our study might also apply to surgeries that are still routinely done with a tourniquet. Therefore, the surgeon should always critically evaluate whether the advantage of a tourniquet application (lower intraoperative blood loss) outweighs the disadvantages (higher need for analgesics, more pronounced IRI).

## Figures and Tables

**Table 1:** Statistical parameters of groups A and B and their *p*-values.

Parameter	Group	Mean Value	SD ^a^	Range	*p*
Tourniquet time	A	30.1 min	5.67	16–44 min	<0.0001
	B	113.2 min	11.23	90–127 min	
Age	A	67.7 y	11.02	47–86 y	0.74
	B	66.5 y	11.18	44–79 y	
BMI	A	29.45 kg/m^2^	5.86	20.1–42.70 kg/m^2^	0.29
	B	31.51 kg/m^2^	6.26	23.5–40.06 kg/m^2^	
ASA	A	2.70	0.56	2–4	0.96
	B	2.71	0.59	2–4	
Tourniquet pressure	A	302.6 mmHg	30.48	270–350 mmHg	0.08
	B	318.8 mmHg	25.22	250–350 mmHg	
Systolic BP before tourniquet	A	114.8 mmHg	17.42	90–145 mmHg	0.09
	B	125.9 mmHg	22.86	90–165 mmHg	
Duration of surgery	A	146.9 min	33.34	90–232 min	0.94
	B	147.8 min	40.33	100–258 min	

^a^ Standard deviation (SD), range, and *p*-values calculated by unpaired *t*-test.

**Table 2:** Statistical parameters of groups A and B, non-continuous variables.

Parameter	Group	Variable a	Variable b	*p* ^b^
Sex	A	10 female	13 male	0.99
	B	8 female	9 male	
Tranexamic Acid	A	10 yes	13 no	0.32
	B	4 yes	13 no	
Type of anesthesia	A	6 Spinal	17 General	0.71
	B	3 Spinal	14 General	

^b^*p*-values calculated by Fisher’s exact test.

## Data Availability

The datasets generated and/or analyzed during the current study are not publicly available due to sensitive patient information, but are available from the corresponding author on reasonable request.

## Supplementary Materials

The following supporting information can be downloaded at: https://www.mdpi.com/article/10.3390/jcm15072675/s1, Supplementary File S1a: CONSORT Checklist [30]; Supplementary File S1b: CONSORT Flow diagram [30]; Supplementary File S2: Statistical Parameters; Supplementary File S3: total amount of morphine equivalent; Supplementary File S4: Effect estimates, 95% CI; Supplementary File S5: C3a; Supplementary File S6: C5a; Supplementary File S7: PAI-1 tPA; Supplementary File S8: Fetuin A; Supplementary File S9: TAT; Supplementary File S10: CK-MM; Supplementary File S11: D-Dimers; Supplementary File S12: M-CSF; Supplementary File S13: IP10; Supplementary File S14: sC5b-9; Supplementary File S15: MIP-1α; Supplementary File S16: 48-plex; Supplementary File S17: raw concentrations.

## Abbreviations

ANOVA	Analysis of variance
ASA-score	American Society of Anesthesiologists score
beta-NGF	Beta-nerve growth factor
bFGF	Basic fibroblast growth factor
BL	Baseline
BMI	Body mass index
BP	Blood pressure
C3a	Complement factor 3a
C5a	Complement factor 5a
CK-MM	Creatine kinase muscle type
CRP	C-reactive protein
CTAK	Cutaneous T-cell attracting chemokine
DVT	Deep vein thrombosis
ELISA	Enzyme-linked immunosorbent assay
G-CSF	Granulocyte-colony stimulating factor
GM-CSF	Granulocyte-macrophage colony-stimulating factor
GRO-alpha	Growth-regulated protein alpha
HGF	Hepatocyte growth factor
IFN-alpha2	Interferon alpha 2
IFN-gamma	Interferon gamma
IL	Interleukin
IP10	Interferon γ-induced protein
IRI	Ischemia/reperfusion injury
Lc	Leukocyte
LIF	Leukemia inhibitory factor
MCP	Monocyte-chemotactic protein
M-CSF	Macrophage colony-stimulating factor
MIF	Macrophage migration inhibitory factor
MIG	Monokine induced by gamma interferon
MIP	Macrophage inflammatory protein
NRS	Numeric rating scale
NSAID	Non-steroidal anti-inflammatory drug
PAI-I/tPA complexes	Complexes of plasminogen activator inhibitor 1 (PAI-I) with tissue-type plasminogen activator (tPA)
PCIA	Patient-controlled intravenous analgesia
PDGF-BB	Platelet-derived growth factor subtype BB
PKA	Protein kinase A
RANTES	Regulated on activation, normal T-cell expressed and secreted
RCT	Randomized controlled trial
sC5b-9	Soluble terminal complement complex
SCF	Stem cell factor
SCGF-beta	Stem cell growth factor beta
SDF-1alpha	Stromal cell-derived factor 1 alpha
TAT	Thrombin–antithrombin complexes
TKA	Total knee arthroplasty
TNF	Tumor necrosis factor
TRAIL	Tumor necrosis factor-related apoptosis-inducing ligand
VEGF	Vascular endothelial growth factor
VTE	Venous thromboembolism
