# Supplementary material for: Tourniquet Duration and Early Clinical and Biomarker Outcomes in Total Knee Arthroplasty: A Comparative Cohort Study
Source: J Clin Med. 2026 Apr 1;15(7):2675. doi: 10.3390/jcm15072675 (PMC13074193; doi:10.3390/jcm15072675)
Supplement: Supplementary file 1 [file jcm-15-02675-s001.zip › Supplementary File S13 (IP10).pdf]

Supplementary File no. S13: IP10

A: Change of IP10 plasma levels between 4 h and 48 h.

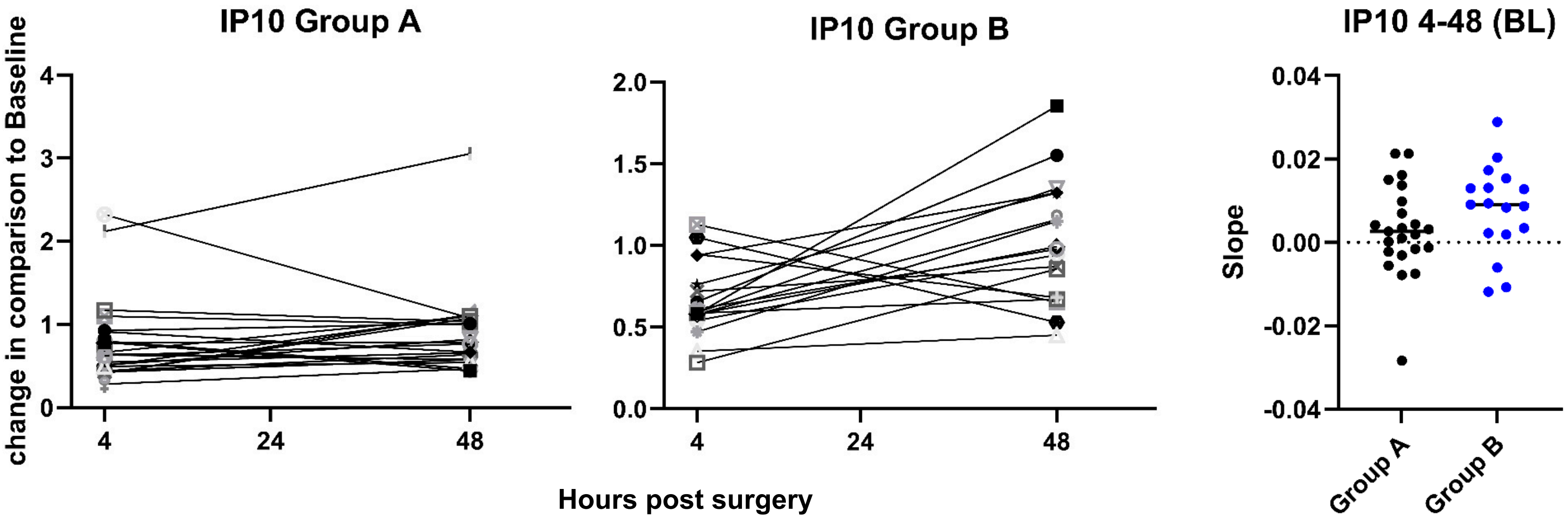

Changes of IP10 plasma levels between 4 h and 48 h after the operation are shown for Group A (left panel) and Group B (middle panel). Values are fold-changes normalized to baseline (BL). The slopes for the change between 4 h and 48 h are plotted on the right panel, with indication of mv, showing no significant higher in- or decrease of the IP10 levels in Group B as compared to Group A, p=0.15, calculated by unpaired t-test. BL: Base Line

B: Change of IP10 plasma levels between 24 h and 48 h.

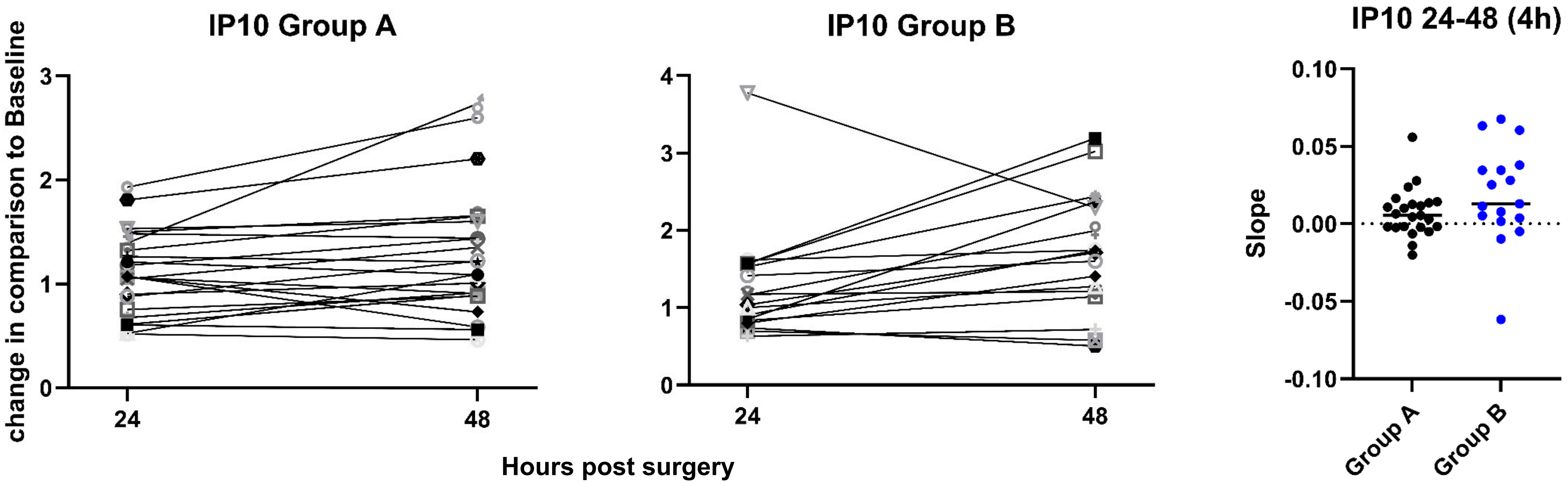

Changes of the plasma levels of IP10 between 24 h and 48 h after the operation are shown for Group A (left panel) and Group B (middle panel). Values are fold-changes normalized to 4 h. The slopes for the change between 24 h and 48 h are plotted on the right panel with indication of the mv, showing no significant higher in- or decrease of the IP10 levels in Group B as compared to Group A, p=0.13, calculated by unpaired t-test.
