# Supplementary material for: Tourniquet Duration and Early Clinical and Biomarker Outcomes in Total Knee Arthroplasty: A Comparative Cohort Study
Source: J Clin Med. 2026 Apr 1;15(7):2675. doi: 10.3390/jcm15072675 (PMC13074193; doi:10.3390/jcm15072675)
Supplement: Supplementary file 1 [file jcm-15-02675-s001.zip › Supplementary File S16 (48-plex).pdf]

Supplementary File no. S16: Cytokine 48-plex

CTAK (BL)

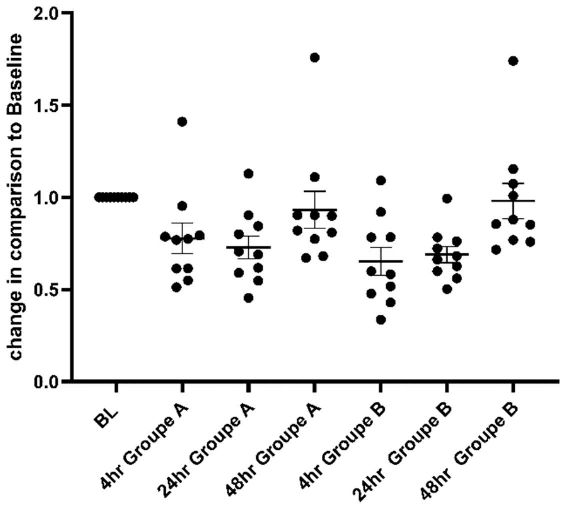

Eotaxin (BL)

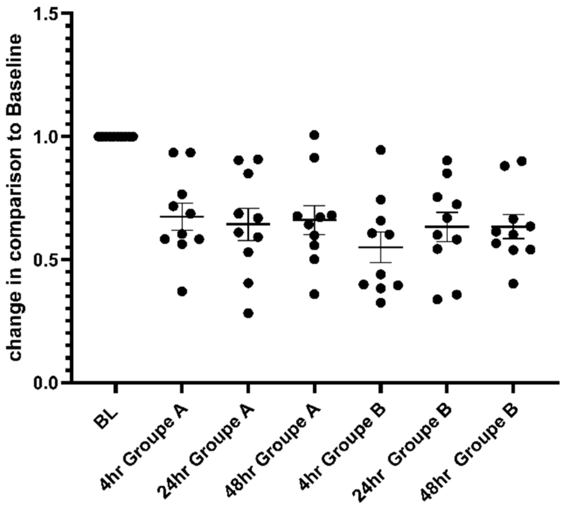

bFGF (BL)

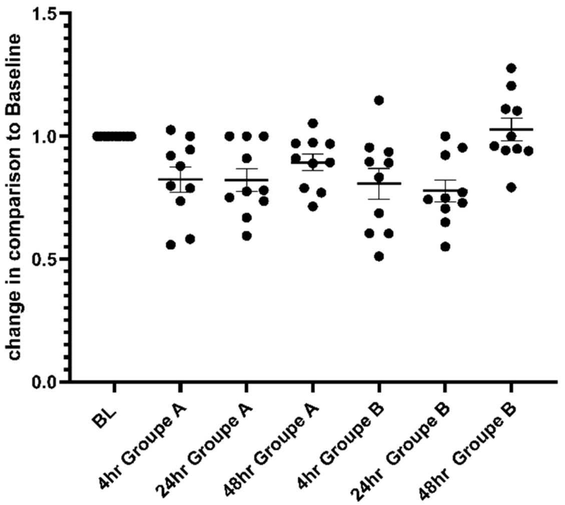

G-CSF (BL)

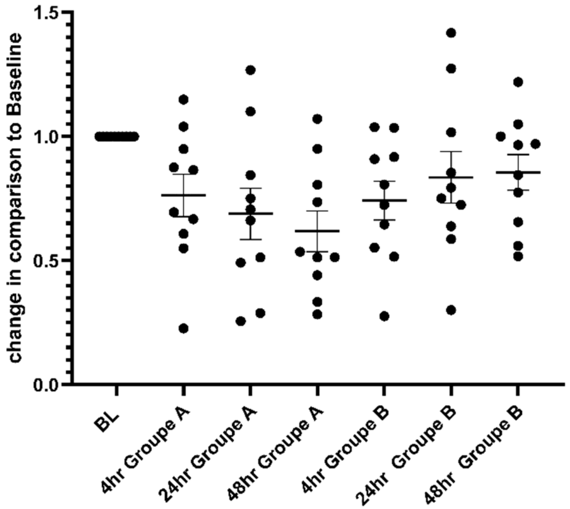

GRO-alpha (BL)

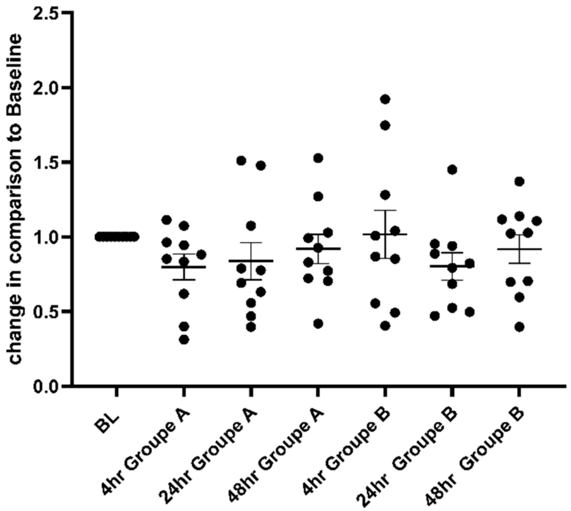

GM-CSF (BL)

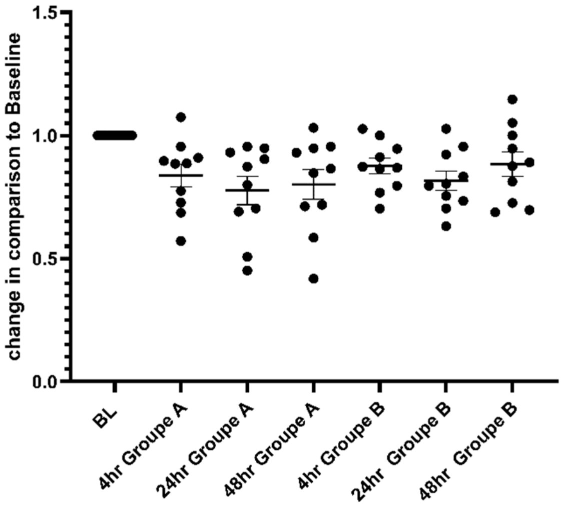

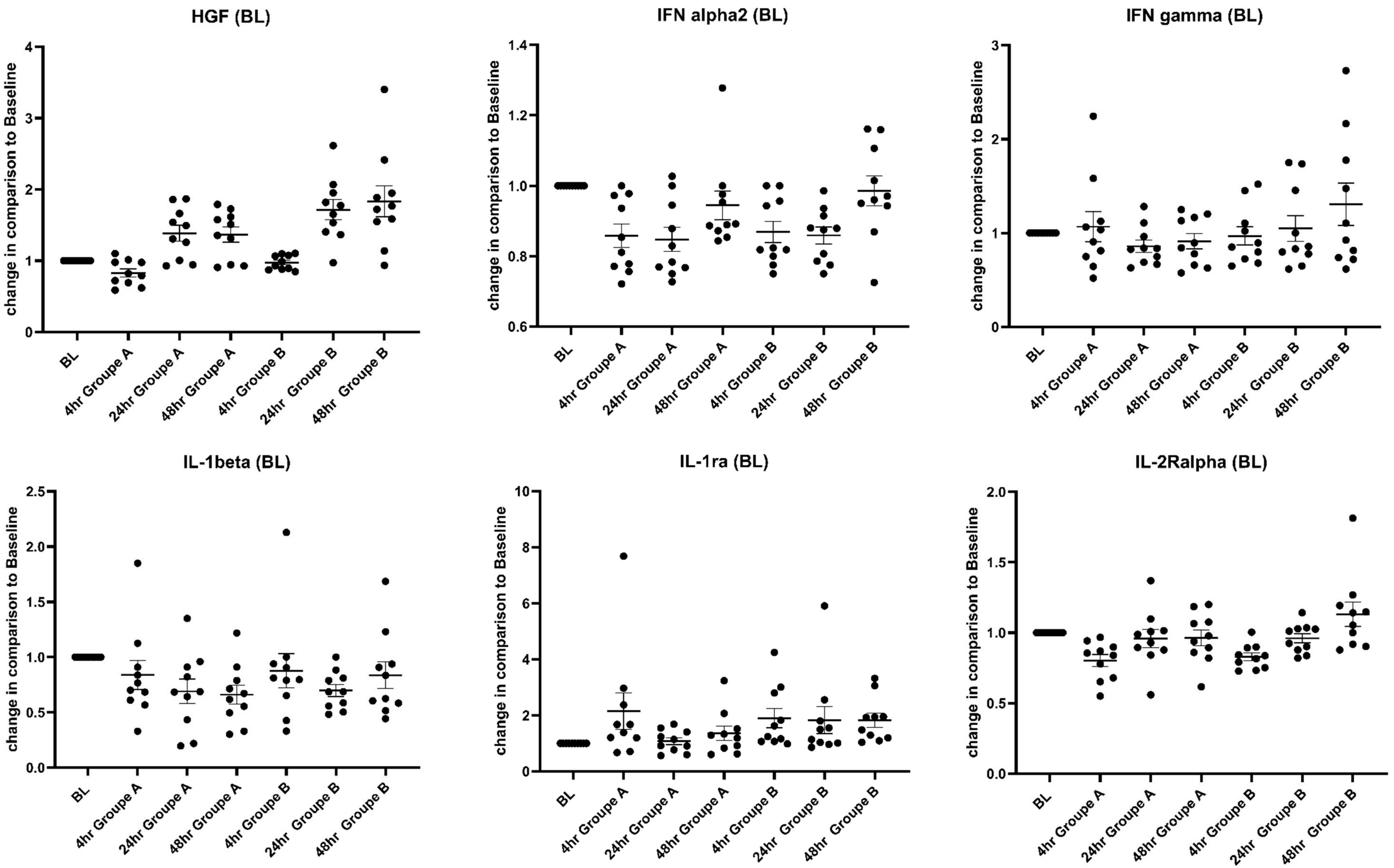
HGF (BL)
IFN alpha2 (BL)
IFN gamma (BL)
IL-1beta (BL)
IL-1ra (BL)
IL-2Ralpha (BL)
change in comparison to Baseline
BL
4hr Groupe A
24hr Groupe A
48hr Groupe A
4hr Groupe B
24hr Groupe B
48hr Groupe B

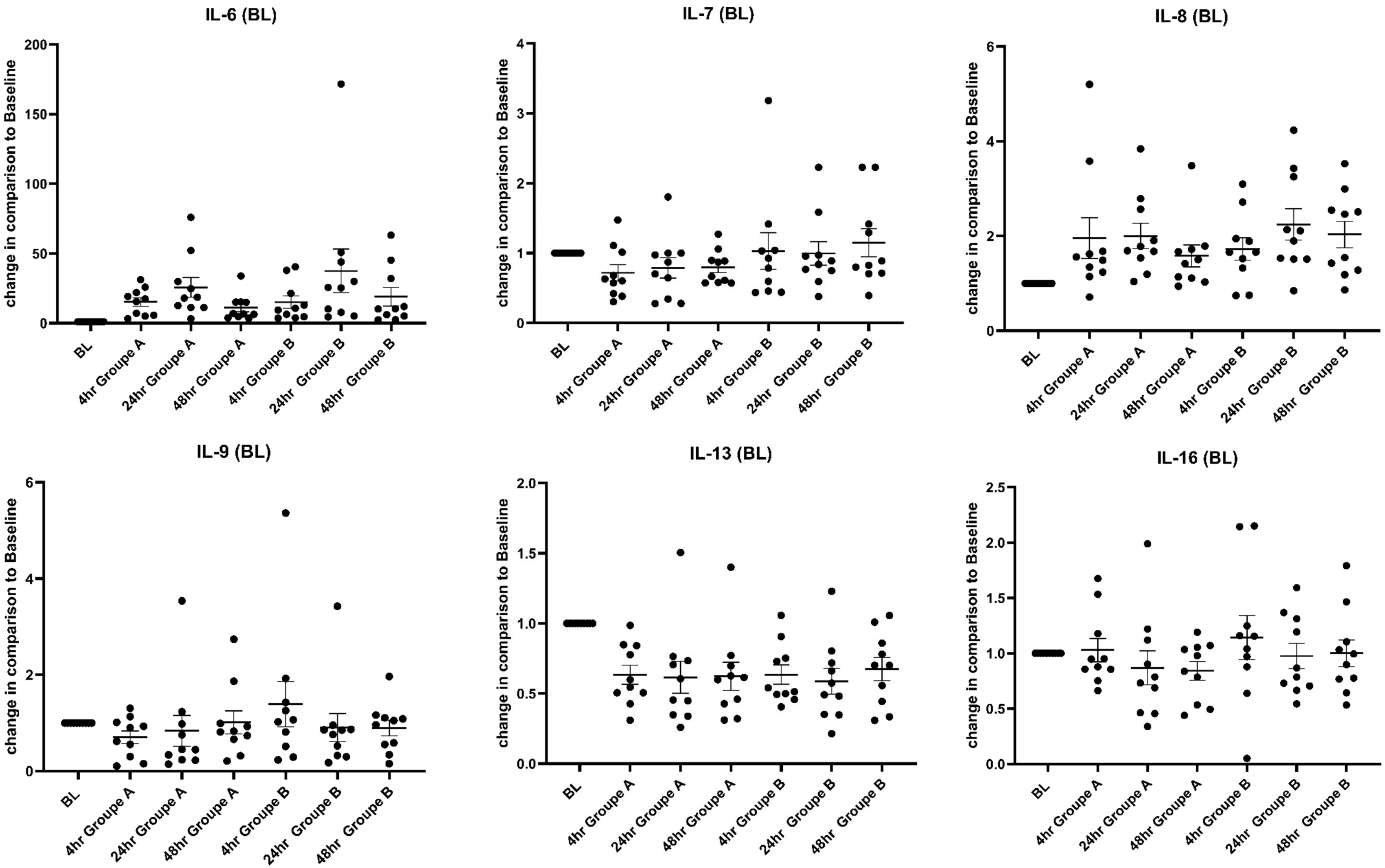
IL-6 (BL)
IL-7 (BL)
IL-8 (BL)
IL-9 (BL)
IL-13 (BL)
IL-16 (BL)
change in comparison to Baseline
BL
4hr Groupe A
24hr Groupe A
48hr Groupe A
4hr Groupe B
24hr Groupe B
48hr Groupe B
0
50
100
150
200
1
2
3
4
6
0.0
0.5
1.0
1.5
2.0
2.5

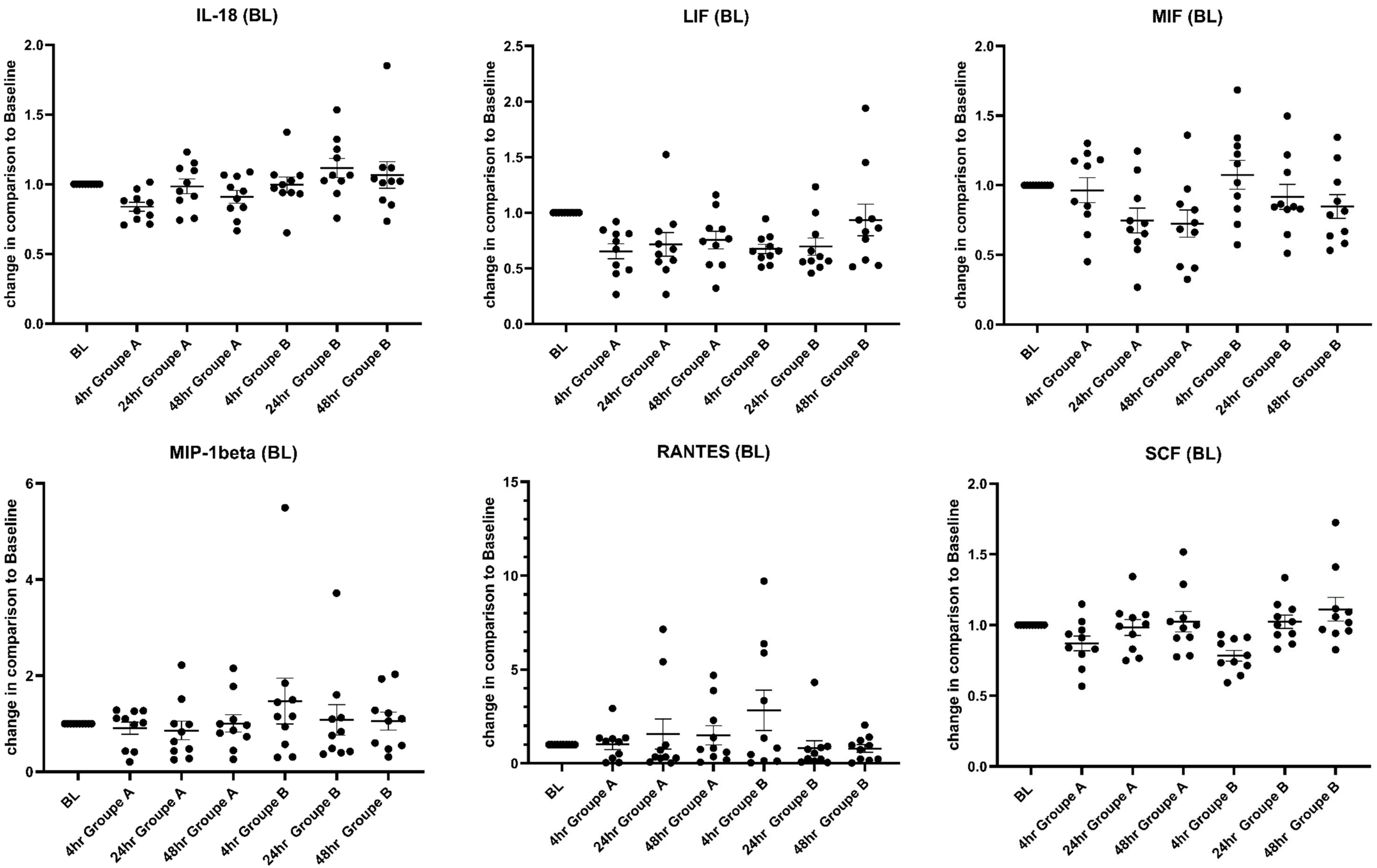
IL-18 (BL)
LIF (BL)
MIF (BL)
MIP-1beta (BL)
RANTES (BL)
SCF (BL)
change in comparison to Baseline
BL
4hr Groupe A
24hr Groupe A
48hr Groupe A
4hr Groupe B
24hr Groupe B
48hr Groupe B

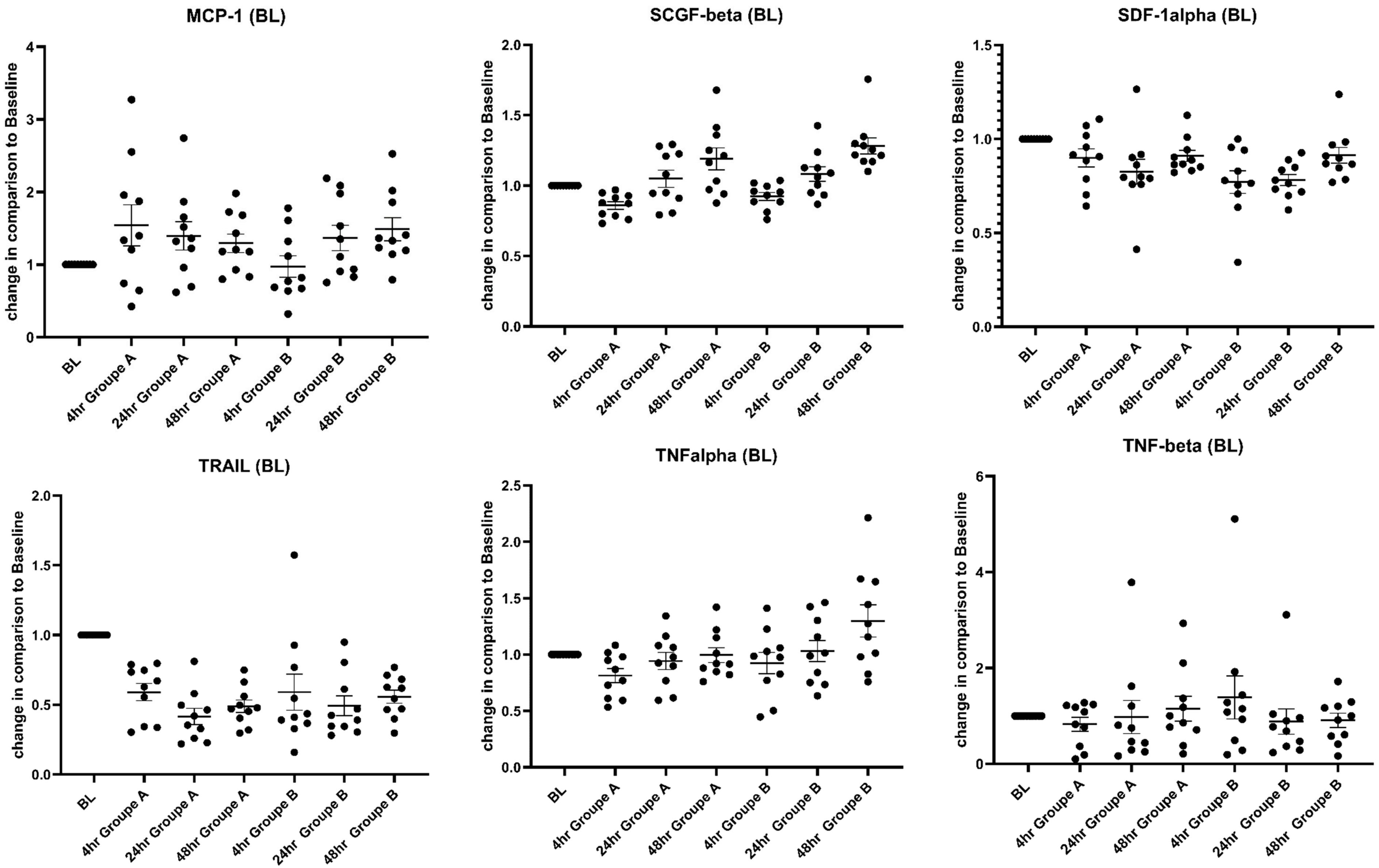
MCP-1 (BL)
SCGF-beta (BL)
SDF-1alpha (BL)
TRAIL (BL)
TNFalpha (BL)
TNF-beta (BL)
change in comparison to Baseline
BL
4hr Groupe A
24hr Groupe A
48hr Groupe A
4hr Groupe B
24hr Groupe B
48hr Groupe B

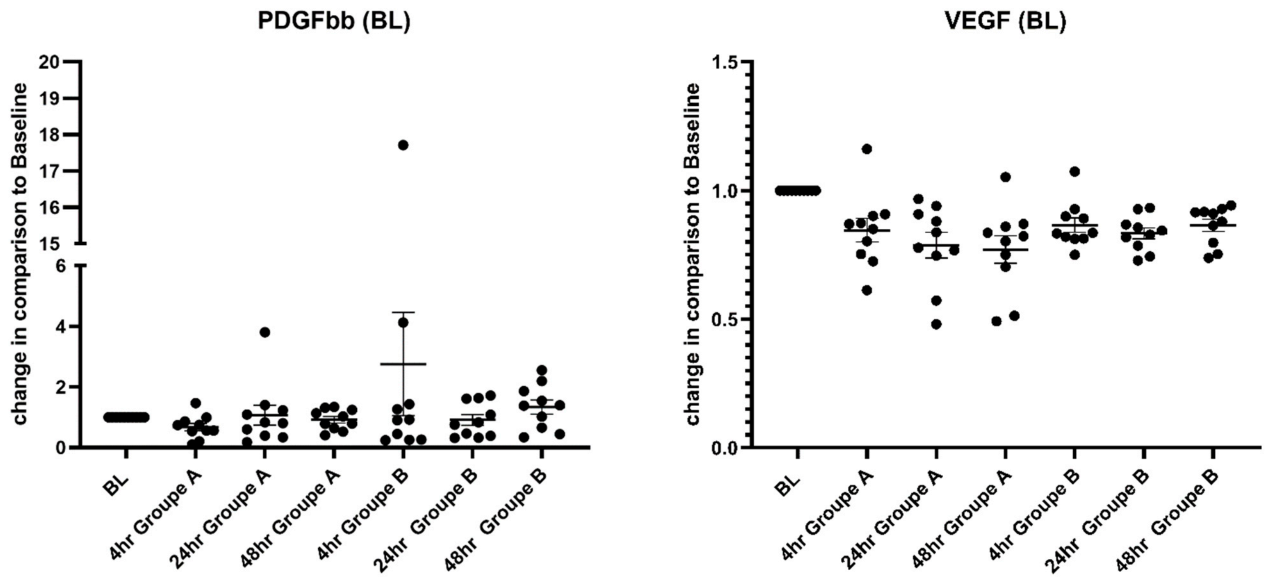

Changes of the plasma levels of the above named markers between 4 h and 48 h after the operation are shown for Group A and Group B for patient 1 - 20. Values are fold-changes normalized to BL with indication of the mv. There were no significant differences, so that testing was not continued. Calculations were done with the one way ANOVA. BL: Base Line.
