# Supplementary material for: Tourniquet Duration and Early Clinical and Biomarker Outcomes in Total Knee Arthroplasty: A Comparative Cohort Study
Source: J Clin Med. 2026 Apr 1;15(7):2675. doi: 10.3390/jcm15072675 (PMC13074193; doi:10.3390/jcm15072675)
Supplement: Supplementary file 1 [file jcm-15-02675-s001.zip › Supplementary File S3 (total amount of morphine equivalent).pdf]

Supplementary file no. S3: Total amount of morphine equivalent for each group

Group	Patient	Equipotent doses of per os morphine p.o. + PCIA [mg]	Group	Patient	Equipotent doses of per os morphine p.o. + PCIA [mg]
A	1	67.5	B	1	161.84
A	2	52.5	B	2	68.91
A	3	285	B	3	131
A	4	142.5	B	4	210
A	5	142.5	B	5	15.4
A	6	142.5	B	6	7.5
A	7	60	B	7	111.8
A	8	0	B	8	203.6
A	9	82.5	B	9	52.5
A	10	75	B	10	112.5
A	11	150.00	B	11	291.90
A	12	105.00	B	12	201.30
A	13	112.50	B	13	90.00
A	14	22.50	B	14	0.00
A	15	37.50	B	15	37.50
A	16	4090.00	B	16	37.50
A	17	157.50	B	17	72.20
A	18	22.50			
A	19	75.00			
A	20	30.00			
A	21	67.50			
A	22	142.50			
A	23	15.00			
