# Supplementary material for: Tourniquet Duration and Early Clinical and Biomarker Outcomes in Total Knee Arthroplasty: A Comparative Cohort Study
Source: J Clin Med. 2026 Apr 1;15(7):2675. doi: 10.3390/jcm15072675 (PMC13074193; doi:10.3390/jcm15072675)
Supplement: Supplementary file 1 [file jcm-15-02675-s001.zip › Supplementary File S7 (PAI-1 tPA).pdf]

**Supplementary File no. S7: PAI1tPA**

**A: Change of PAI1tPA plasma levels between 24 h and 48 h.**

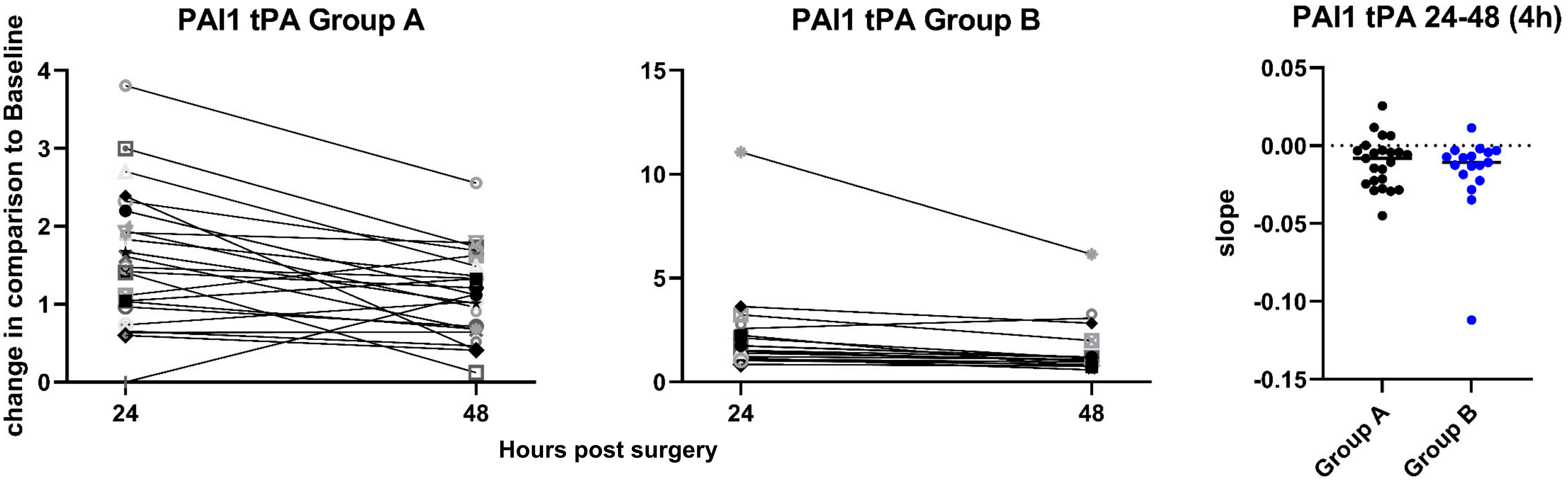


Changes of the plasma levels of PAI1tPA between 24 h and 48 h after the operation are shown for Group A (left panel) and Group B (middle panel). Values are fold-changes normalized to 4 h. The slopes for the change between 24 h and 48 h are plotted on the right panel with indication of the mv, showing no significant higher in- or decrease of the PAI1tPA levels in Group B as compared to Group A, p=0.38, calculated by unpaired t-test.
