# Supplementary figures and images for: Tourniquet Duration and Early Clinical and Biomarker Outcomes in Total Knee Arthroplasty: A Comparative Cohort Study
Source: J Clin Med. 2026 Apr 1;15(7):2675. doi: 10.3390/jcm15072675 (PMC13074193; doi:10.3390/jcm15072675)

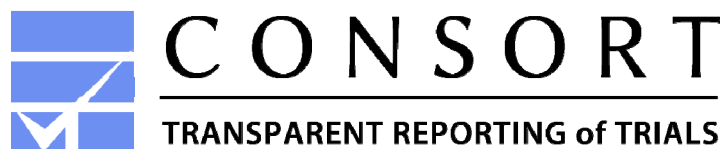

### CONSORT 2010 Flow Diagram

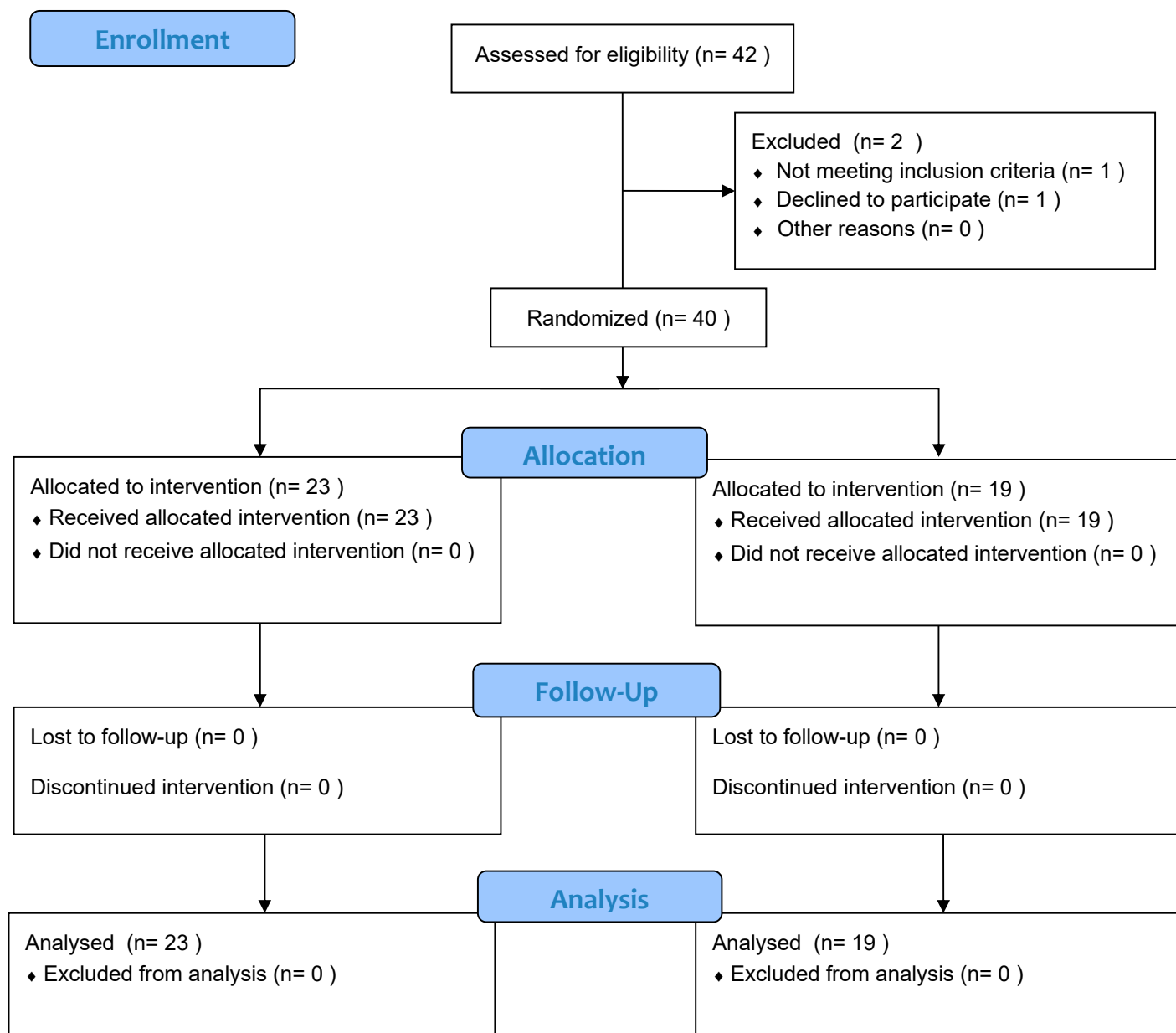

Supplement: Supplementary file 1 [file jcm-15-02675-s001.zip › Supplementary file S1b (CONSORT flow diagram).pdf]
